# Parallel Ictal-Net, a Parallel CNN Architecture with Efficient Channel Attention for Seizure Detection

**DOI:** 10.3390/s24030716

**Published:** 2024-01-23

**Authors:** Gerardo Hernández-Nava, Sebastián Salazar-Colores, Eduardo Cabal-Yepez, Juan-Manuel Ramos-Arreguín

**Affiliations:** 1 Faculty of Engineering, Autonomous University of Querétaro, Queretaro 76140, Mexico; gerardohn.uam@gmail.com (G.H.-N.); jsistdig@yahoo.com.mx (J.-M.R.-A.); 2 Research Department, Centro de Investigaciones en Óptica A.C., Guanajuato 37150, Mexico; 3 Multidisciplinary Studies Department, Campus Yuriria, University of Guanajuato, Guanajuato 38954, Mexico; educabal@ugto.mx

**Keywords:** CNN, CWT, efficient channel attention, seizure detection

## Abstract

Around 70 million people worldwide are affected by epilepsy, a neurological disorder characterized by non-induced seizures that occur at irregular and unpredictable intervals. During an epileptic seizure, transient symptoms emerge as a result of extreme abnormal neural activity. Epilepsy imposes limitations on individuals and has a significant impact on the lives of their families. Therefore, the development of reliable diagnostic tools for the early detection of this condition is considered beneficial to alleviate the social and emotional distress experienced by patients. While the Bonn University dataset contains five collections of EEG data, not many studies specifically focus on subsets D and E. These subsets correspond to EEG recordings from the epileptogenic zone during ictal and interictal events. In this work, the parallel ictal-net (PIN) neural network architecture is introduced, which utilizes scalograms obtained through a continuous wavelet transform to achieve the high-accuracy classification of EEG signals into ictal or interictal states. The results obtained demonstrate the effectiveness of the proposed PIN model in distinguishing between ictal and interictal events with a high degree of confidence. This is validated by the computing accuracy, precision, recall, and F1 scores, all of which consistently achieve around 99% confidence, surpassing previous approaches in the related literature.

## 1. Introduction

More than 70 million people worldwide have epilepsy, a non-transmissible, chronic neurological disorder, which does not distinguish among age, sex, or race. Epilepsy is characterized by non-induced convulsions occurring in an intermittent and random way. In 2014, the International League Against Epilepsy (ILAE) designated epilepsy as a brain disease characterized by any of the following conditions: occurrence of at least two non-provoked seizures, 24 h apart, one non-provoked crisis with a minimal probability of 60% of suffering new seizures through the following 10 years, or having an epilepsy syndrome diagnosis [1].

During an epileptic seizure, transient indicators or symptoms appear because of excessive or simultaneous abnormal neural activity. The symptoms can include temporal disorientation, absent lapses, loss of consciousness, psychic symptoms (e.g., dread, anxiety, and déjà vu), as well as sudden and uncontrollable muscular-contraction movements of the body extremities. Suffering from this disease restricts people in different ways and affects families by disrupting their daily lives, making them the target of social and workplace discrimination, service denial, social stigma, and, as a consequence, resulting in depression [1]. Therefore, it is necessary to develop highly reliable assistant tools for carrying out a timely diagnosis of epilepsy to diminish social and emotional affectations regarding patients.

Input data are key elements in the development of aiding tools for the detection of epileptic surges; hence, most works concerning this subject use electroencephalography (EEG) signals as an information source, since they provide essential physiological indicators to diagnose, treat, and trace epilepsy [2]. The EEG is an electrical signal in the order of microvolts [µV], which reflects the brain’s activity by placing superficial electrodes on the scalp or intracranial ones directly on the brain. An EEG signal shows different stages that can be used for detecting epileptic phases: preictal, a brief time before an epileptic seizure; ictal, the middle stage of a stroke; postictal, the state just after an epileptic attack; and interictal, the lapse of time between consecutive epileptic events [3].

Previous works have predicted, detected, and classified epileptic incidents, looking to help patients with this disease, by utilizing artificial intelligence (AI), specifically deep learning (DL). The most-employed approaches involve support vector machines (SVMs) [4], k-nearest neighbors (KNNs) [5], Bayesian networks (BNs) [6], decision trees (DTs) [7], artificial neural networks (ANNs) [8], long short-term memories (LSTMs) [9], and convolutional neural networks [10]. Distinct characterization methods of the EEG signal time–frequency domain utilizing the discrete wavelet transform (DWT). Furthermore, some works utilize images generated by means of the Gramian angular field transform to classify the EEG signal.

In this work, the EEG signals are characterized in the time–frequency domain utilizing the continuous wavelet transform (CWT). The CWT is particularly effective for characterizing EEG signals in the time–frequency domain, especially given the non-stationary nature of EEG data. Unlike other techniques, such as the short-time Fourier transform (STFT), CWT excels in its adaptability to varying frequencies over time, making it well-suited for an EEG analysis. This adaptability allows the CWT to simultaneously analyze both high- and low-frequency components within EEG signals, offering a comprehensive view of brain activities across different frequency bands. Furthermore, the CWT’s high resolution in the time–frequency domain provides a more accurate representation of ictal and interictal events, which are crucial for understanding and diagnosing neurological conditions. The output of the CWT, known as a scalogram, visually represents the variation of frequency components over time in a logarithmic format, thereby offering an insightful visualization of the complex dynamics in EEG signals. These obtained spectrograms are the input to the proposed parallel ictal-net (PIN), which is an architecture that contains neural networks working in parallel, and each neural network has an efficient channel attention module that is described in detail in Section 3.3. The obtained results demonstrate that the proposed PIN can efficiently discern between ictal and interictal events with high certainty, outperforming recently have been employed as input data sources to obtain parameters, such as the average value, variance, standard deviation, root mean square (RMS), power spectral density (PSD), as well as other reference values obtained through the signal’s transformation into the introduced approaches, by computing the performance metrics, accuracy, precision, recall, and F1 scores, which are the most occupied efficacy measurements in the associated literature, obtaining around a 99% confidence value for all of them.

## 2. Related Works

Even though there is a substantial number of works that analyze the Bonn University dataset [11], a publicly available database of EEG signals, most of them display an assortment among all the five subsets, or categories, contained in it. This section analyzes the state of the art in the subject by examining related works in the literature pursuing the goal of achieving the classification of the EEG signals in the Bonn database, specifically those corresponding to the ictal and interictal states, contained in the subsets D and E, respectively, which contain EEG registers taken from the epileptogenic zone. On this subject, in [6], subsets D and E are classified through SVMs, KNNs, and BNs, reaching an effectiveness between 88.60% and 91.16%, depending on the used classification algorithm. The results are obtained through two- and four-fold cross validations. The EEG signals are processed utilizing a radial-basis function to obtain parameters, such as the average, variance, standard deviation, root mean square (RMS), and power spectral density (PSD) mean, to achieve their classification.

In [12], the Bonn University database is classified using SVMs, KNNs, and a DT. The EEG signals are parameterized by applying the DWT and the empirical mode decomposition (EMD), achieving accuracy rates ranging from 60% to 100%. In [13], various algorithms, such as the SVM, ANN, LSTM, among others, were compared for classifying EEG signals in this dataset. In [14], the same database was classified through a convolutional neural network (CNN), which processed images generated via the Gramian angular field transform. This method involved applying the Gramian angular sum field and the Gramian angular difference field to the EEG signal and its corresponding instantaneous power. In [5], machine learning algorithms combined with a fuzzy classifier were employed, achieving over 90% accuracy in classifying the Bonn dataset. An LSTM network was proposed in [15] for the database classification, featuring four layers and trained using a 10-fold cross-validation procedure with 30 epochs and a batch size of 32. The ictal-net, introduced in [16], is a CNN designed to classify EEG signals segmented into 1.6 s windows, achieving a 93.7% accuracy rate. A novel approach for epilepsy detection using a variable-frequency complex demodulation (VFCDM) to obtain high-resolution time–frequency spectra (TFSs) and a CNN has recently emerged [17]. This method, differing from traditional techniques, like the continuous wavelet transform (CWT), has shown promising results in identifying various epilepsy states through the EEG analysis. However, given the excellent outcomes achieved in similar tasks with simpler CNN networks [18], it is a logical progression to explore the combination of the CWT with the CNN for further advancements in this field.

In light of the preceding discussion, the primary objective of this investigation is to conduct a classification of EEG signals derived from patients diagnosed with epilepsy. The aim is to accurately distinguish between ictal and interictal states, utilizing the database of Bonn University. To fulfill this objective, a novel neural network architecture, henceforth referred to as the parallel ictal-net (PIN), is meticulously designed and subsequently applied. The successful achievement of a highly reliable differentiation between ictal and interictal states is anticipated to facilitate the implementation of more efficacious treatment protocols for individuals afflicted by epilepsy, with the ultimate goal of significantly enhancing the quality of life for both the patients and their respective families.

## 3. Theoretical Background

### 3.1. Continues Wavelet Transform

The continuous wavelet transform (CWT) is a mathematical approach employed to deconstruct a continuous or discrete signal into its constituent frequency components. This technique proves invaluable for the analysis of transient signals, as it provides detailed information regarding their variations in both the time and frequency domains. Consequently, this allows for the extraction of meaningful characteristics that remain elusive when other methods, such as the Fourier transform, are utilized [19].

**Figure 1:**
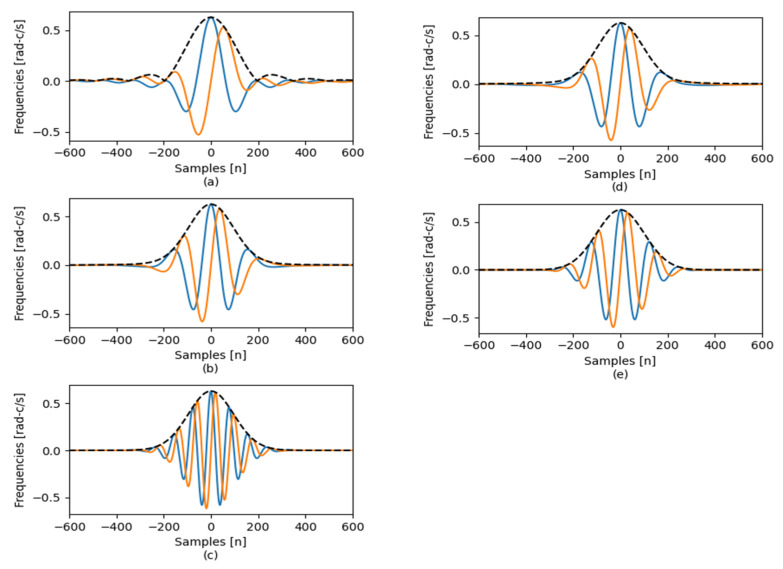
Mother wavelet forms: (**a**) bump, (**b**) complex Mexican hat, (**c**) generalized Morse wavelet, (**d**) Hilbert analytic function of Hermitian hat, (**e**) Morlet ictal signal.

The CWT is implemented for an analyzed signal by utilizing a mother-wavelet function as a basis. Each mother wavelet has specific features, such as waveform and temporal width, which are employed for determining the distinct frequency components in the signal [17]. Figure 1 depicts different mother-wavelet functions.

The signal analysis was performed by scaling the mother-wavelet function and adapting the deduced information in time in such a way that it matched the analyzed signal. The resulting wavelet coefficients provided the frequency contents of the signal for each scale (wavelength width) and location (shifting). A scalogram can be obtained from the wavelet coefficients through further processing, to determine the high-efficiency features and patterns associated with the analyzed signal. 

**Figure 2:**
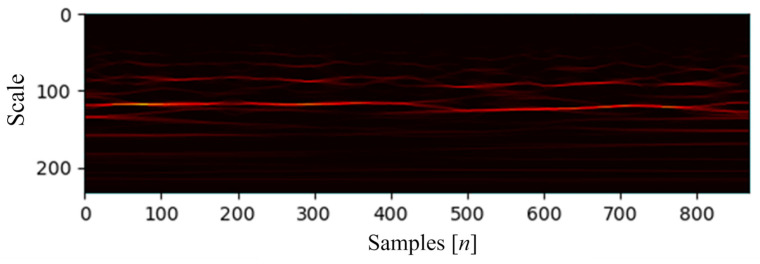
Ictal signal scalogram using the bump mother-wavelet function.

A scalogram is a graphical representation of a signal’s frequency overtime. It is usually employed for image and signal processing to analyze time and space variations [20,21]. Figure 2 illustrates an 868 point ictal signal scalogram.

### 3.2. Deep Learning and the CNN

Deep learning (DL) is a subarea of artificial intelligence (AI), which usually works with large datasets for statistical applications and predictive models. In recent years, there has been a broad number of areas where DL has shown its usefulness, including image classification [22], natural language processing [23], financial tasks [24], and the energy sector [25], among many others. The most outstanding aspect of DL is that it is an automatic self-learning AI technique, which has proven to be highly effective in solving complex problems. In this regard, an artificial neural network (ANN) is a computational model that consists of artificial neuron sets, which carry out sequential connections among themselves, grouped into structures known as layers. Usually, an ANN has an input layer, one or more in-between layers of neurons, denominated hidden layers, with activation functions that deliver an output.

Deep learning is based on the implementation of a deep ANN. CNNs are a category of ANN used in this field, which were proposed in 1998 by LeCun et al. [26]. CNNs are specially designed for image identification and classification purposes, where the initial layers learn about simple features, such as lines, curves, and contours to recognize the image complex details of the deepest layers.

CNNs can be described through five main operations [26]: (1) convolution is used for identifying the patterns and features composing the analyzed information. This operation is performed utilizing kernels, which are small-sized matrices applied to the analyzed images for emphasizing some of their features. (2) Grouping is employed for reducing the dimensions of the matrices and vectors computed during the convolutional operation by removing some elements to simplify the information processing. The most used techniques for performing this task are computing either the maximum value of the applied kernel or its average. (3) Activation is the stage where the non-linearity of the analysis is introduced through functions, such as SoftMax, rectified linear unit (ReLU), sigmoid, Gaussian error linear units (GELUs), among many others, which assist the CNNs with learning complex patterns. (4) Normalization improves the network training and its performance by adjusting all the values to follow the same distribution. The most used approach for carrying out this procedure is batch normalization. Classification is the final stage of a CNN where labels are assigned to input images according to the model learned by the network.

### 3.3. Efficient Channel Attention

An attention module is a supplementary layer that allows the ANN architecture to focus on specific information defined during the processing; in other words, it grants the ANN the ability of paying attention to certain patterns without assigning fixed weights or handling all the data in a similar manner, allowing it to improve the efficiency and performance of the process [27]. At present, attention modules are widely used [28,29,30]; for instance, in [28], a self-attention module was introduced to endorse the process for distinguishing among the suitability of different input tokens during a data series analysis.

There are several attention mechanisms, for instance, soft attention, attention autoencoders, and non-local attention, among others. Each one has specific characteristics that make them suitable for different applications. Some areas where attention mechanisms are widely utilized are natural language processing, computer vision, and voice recognition [31,32,33]. In this work, after an extensive ablation study, an efficient channel attention module was employed because it provided the best performance results against a channel attention block, a pixel attention block, and not using an attention mechanism.

As stated before, CNNs consist of convolutional layers where filters are applied to the input data for obtaining multi-channel characteristic maps as outputs, with each layer providing a different feature from the processed data. Hence, efficient channel attention modules allow the convolutional architecture to focus on the most-relevant elements by adding an additional layer to the CNNs. Attention layers learn selectively utilizing a training function, which picks out or discards some channels in order to attain more effective predictions, reducing the computational cost, since irrelevant components for the required task are disregarded, allowing it to produce a more efficient convolutional architecture.

The efficient channel attention technique has been utilized for tasks, such as natural language processing, object detection, and image classification [34,35], to improve the efficiency and performance of the defined models. It can be implemented in different ways, for instance, the squeeze-and-excitation module described in [27], which consists of two main operations. The compression operation reduces the spatial dimensionality of the feature map, through global mean grouping, delivering a statistical vector for each channel, whereas the excitation process utilizes this statistical vector, a fully connected layer, and a sigmoid activation function to adjust the characteristics for the channel output as:

(1) $$f_{c}=sigmoid\left\{W_{2}\left[ReLu\left(W_{1}y\right)\right]\right\}$$

where y is the obtained statistical vector for each channel, W1 and W2 are learned weights, and $f_{c}$ is the excitation operation output function for channel c. The module outcome is obtained by multiplying the input feature map by the excitation operation output function, $f_{c}$.

In a different scenario, the efficient channel attention module (ECA), described in [27], utilizes a CNN, instead of a fully connected layer, to learn the attention weights for each channel, which are generated through the excitation operation output function, to scale the feature map at the input by:

(2) $$f_{c}=sigmoid\left[conv\left(y\right)\right]$$

where conv is the convolutional layer.

### 3.4. The Bonn Database

The database utilized in this work was sourced from the University of Bonn [36]. It comprises five sets, labeled A to E, each containing 100 single-channel EEG recordings, each lasting 23.6 s. These signals were recorded at a sampling frequency of 173.61 Hz, using a pass-band filter ranging from 0.53 to 40 Hz.

The database batches were distributed as:

- Group A: five healthy subjects in a relaxation state with eyes closed.
- Group B: five healthy subjects in a relaxation state with eyes open.
- Group C: five pathological subjects free from seizures, whose EEG registers were obtained from the hippocampus formation in the brain’s opposite hemisphere.
- Group D: five pathological subjects free from seizures, whose EEG registers were obtained from the epileptogenic zone.
- Group E: five pathological subjects with epileptic seizure activity.

The primary aim of the proposed parallel ictal-net (PIN) architecture is to differentiate between ictal and interictal EEG signals. For this purpose, only datasets D and E are utilized in this study, as they uniquely consist of recordings from the epileptogenic zone in the same hemisphere.

**Figure 3:**
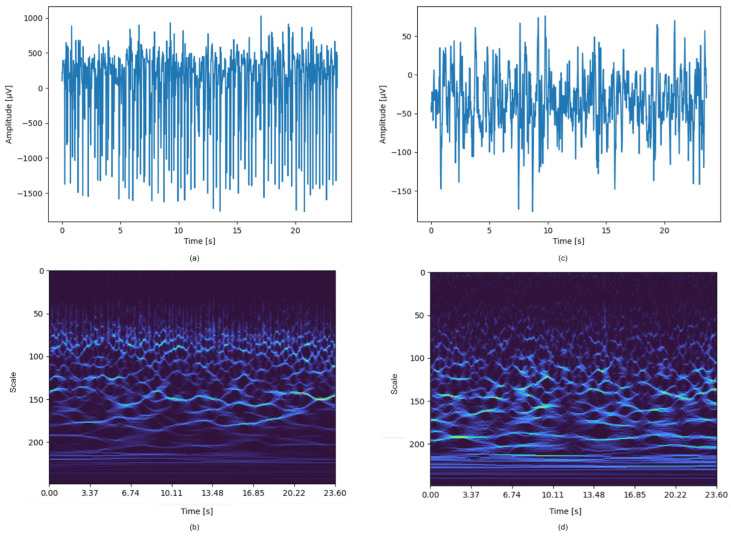
EEG Signals and corresponding scalograms using the bump wavelet. (**a**) EEG signal during an ictal state; (**b**) scalogram derived from the ictal signal; (**c**) EEG signal during an interictal state; (**d**) scalogram generated from the interictal signal.

Figure 3 illustrates examples of EEG signals from the Bonn database, accompanied by their respective scalograms generated through the application of the continuous wavelet transform (CWT). It is crucial to highlight that discerning straightforward patterns within these scalograms is challenging, thereby necessitating the use of advanced techniques founded on artificial intelligence models to effectively interpret them. This figure underscores the complexity of the EEG data and the value of AI-driven analytical approaches in this domain.

## 4. Parallel Ictal-Net Integration

The parallel ictal-net (PIN) synthesis was performed considering six sets of variables, which included (a) the mother-wavelet function election, where the generalized Morse wavelet (gmw), the Morlet, the bump, the complex Mexican hat (cmhat), and the Hilbert analytic function of Hermitian hat (hhhat) were considered. (b) The sampling frequency (Fs), used to compute the scalograms, which adopted either a value of 173.61 or 0. (c) The number of samples on each window, which changed from 174 to 2083. (d) The overlap percentage between consecutive windows, which can be zero, one quarter, one third, or half a window. (e) The learning rate of the neural network training, which takes a value of 0.01, 0.001, or 0.0001. (f) The optimizer type, which can be an Adam optimizer or a stochastic gradient descent (SGD). All these specifications are shown in Table 1.

Table 1 shows a total of 28 variables, distributed across six adjustable hyperparameters, each integral for the accurate recognition of epileptic events. To ensure the robustness of our results, we employed the stratified 10-fold cross-validation technique to evaluate each combination of these hyperparameters systematically. The integration process of the parallel ictal-net (PIN) was structured as several key stages:

(a) Signal pre-processing: this initial stage involved segmenting the acquired EEG signal into windows. For each window, a scalogram was computed and normalized using the continuous wavelet transform (CWT), preparing the data for subsequent analyses.

(b) Pre-training: before the main training phase, the PIN underwent a pre-training stage. This step was crucial for fine-tuning the network’s parameters, ensuring that it was primed for an optimal performance during the main training process.

(c) PIN training: the core of the integration process, the training stage of the PIN, was multifaceted. It included transfer learning, which leveraged the pre-trained models to enhance the learning efficiency; block-coding, which systematically organized the network’s layers; and the actual training phase, where the network learnt to classify EEG signals into different epileptic states.

(d) PIN evaluation: the final stage was the comprehensive evaluation of the trained PIN. This phase assessed the network’s performance in accurately detecting and classifying epileptic events, ensuring its effectiveness and reliability as a diagnostic tool.

### 4.1. Signal Pre-Processing

The EEG signals in the database were already filtered; therefore, signal pre-processing consisted of resampling the signals according to the desired window length (i.e., number of samples) and the overlap percentage between consecutive windows. For instance, the pre-processed signals introduced to the PIN were arranged into 868 sample windows, with a 33.3% overlap, delivering seven windows for each signal, which were treated utilizing the CWT implemented in Python, utilizing the ssqueezepy library [35], to be entered into a graphics processing unit (GPU), exponentially decreasing the computation time. There were two alternatives to perform the CWT calculation: in the first one, the sampling frequency was provided, and in the second one, it was not; however, the mother-wavelet function must always be specified.

Once the CWT coefficients, $C_{i}$, were obtained, their corresponding absolute values were computed and normalized, $C_{i\_N}$, as described in Equation (3), to estimate the scalogram, which was processed through the proposed PIN:

(3) $$C_{i\_N}=\frac{C_{i}-C_{min}}{C_{max}-C_{min}}$$

where $C_{min}$ and $C_{max}$ represent the minimum and maximum absolute values, respectively, of the computed CWT coefficients.

### 4.2. Pre-Training

The pre-processing stage was added to achieve a greater performance through the proposed architecture, before carrying out the transfer learning for every convolutional block. The pre-processing stage consisted of splitting up the EEG signal into the *n* scalograms composing it; therefore, 180×*n* signals were obtained for teaching the pre-training block, since the overall network required 180 signals to be trained.

The pre-training block was composed of a 2D convolutional layer, an efficient channel attention module, a max-pooling layer, and two neurons with a SoftMax activation function at their outputs. This architecture, which was discussed thoroughly in Section 5.2, was identical to that of the convolutional blocks in the networks from the reviewed literature, which were used for a comparison. The pre-training block was taught in 150 epochs; however, an early stopping callback was implemented to avoid overtraining the network.

### 4.3. Network Training

The main difference among the considered networks was the number of convolutional blocks building them, which depended on the number of scalograms, *n*, from the analyzed signal. Transfer learning was carried out first to assign the weights, obtained during the pre-training phase, to the layers of each convolutional block building the network; then, the network was trained by varying the optimization and learning rate values, keeping the number of epochs fixed to 150 and the batch size set to 32. An early stopping callback was implemented to reduce the training time, improve the network’s performance, and avoid overtraining. The early stopping callback ended the training process when a 100% accuracy was achieved in the training set.

### 4.4. Validation

A 10 k-fold cross-validation was performed to verify each proposed network by computing the *accuracy*, *precision*, *recall*, and *F1 score*, which were the most utilized effectiveness criteria in the literature regarding the identification of ictal and interictal states from the EEG signals of epileptic patients. These performance parameters are computed by Equations (4), (5), (6), and (7), respectively:

(4) $$accuracy=\frac{TP+TN}{TP+TN+FP+FN}$$

(5) $$precision=\frac{TP}{TP+FP}$$

(6) $$recall=\frac{TP}{TP+FN}$$

(7) $$F1\;score=2\times \frac{Precision\times Recall}{Precision+Recall}$$

From (4) to (7), the true positives (TPs) are the ictal events correctly identified; false positives (FPs) correspond to the scalograms incorrectly classified as belonging to ictal episodes; true negatives (TNs) are the interictal windows properly identified; and false negatives (FNs) are the interictal episodes recognized as ictal events by the PIN. The reported value of the performance metrics was obtained by computing the corresponding average from 10 distinct trials.

### 4.5. Computer System

The proposed methodology was used in four distinct computer systems. The first one consisted of four NVIDIA-RTX-3090 GPUs and a RYZEN Threadripper processor 1920X with 128 GB of RAM. The second one was composed of an NVIDIA RTX 3080 GPU and a RYZEN-7 processor 1700X with 48 GB of RAM, and the third one was composed of an NVIDIA-RTX 3060 GPU and a RYZEN-5 processor 5600 G with 48 GB of RAM. Hence, there were seven GPUs, 142 GB of VRAM, and 256 GB of RAM in total. All computer systems functioned under the Ubuntu-22.04 operating system. The ssqueezepy library [37] was used for computing the scalograms through the CWT, whereas the Keras library [38] was utilized for designing, compiling, and training the neural network, utilizing the Python programming language.

## 5. Results

This section shows the obtained results from searching the optimal hyperparameters for the proposed PIN compilation to achieve the best performance for classifying between ictal or interictal events, as well as its architecture. Then, the input signal in the PIN was depicted, and a thorough comparison of the obtained results from the proposed architecture, against those from recently proposed approaches in the state of the art, was performed through the previously described performance metrics.

### 5.1. Hyperparameter Search Results

**Figure 4:**
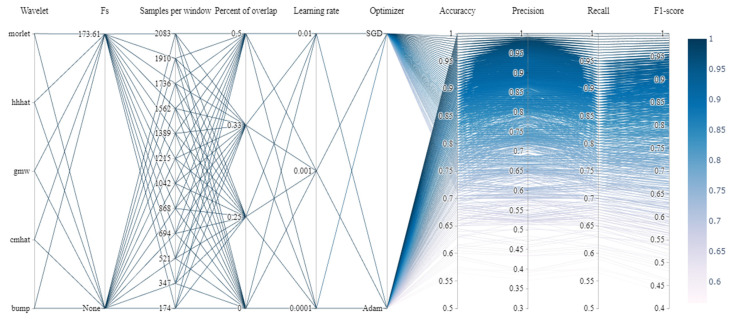
Parallel coordinate plot displaying the tracking of parameters employed in the network design throughout the experimentation process.

A comprehensive assessment of 2880 unique neural network configurations was conducted, taking into account the 28 variables listed in Table 1, which were distributed among the six tunable hyperparameters. This approach ensured a thorough optimization of the proposed PIN architecture. To analyze parameter tuning, a parallel coordinates graphic, as depicted in Figure 4, was utilized. This graphic aided in fine-tuning the parameters to achieve the best quantitative results in terms of the performance metrics commonly used in the related literature.

Figure 4 shows the five different wavelet functions in the first coordinate, followed by the sampling frequency, which can or cannot be defined to compute the CWT; then, the number of samples on each window, the overlap percentage between consecutive windows, the learning rate at which the neural network is trained, the optimizer type, and the four performance metrics to record the efficiency of the 2880 tested architectures are presented. In Figure 4, the lines represent the hyperparameter combination through a heat map, which depends on the obtained performance metric values; the darker the color, the higher the obtained metrics, with 1 corresponding to 100%.

**Figure 5:**
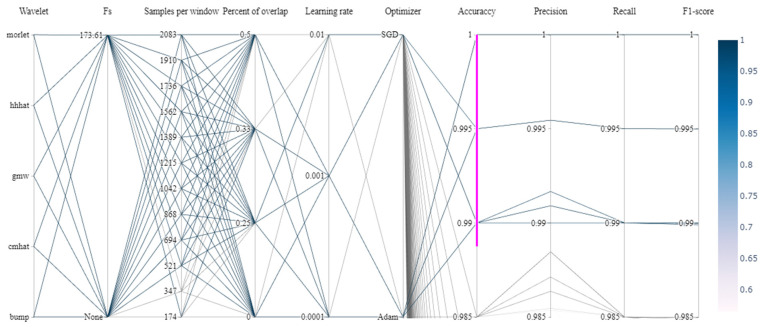
Close up visualizing the architecture’s ictal signal. Network configurations that cross the pink line are those considered to have the best performance.

Of the 2880 tested network configurations, 135 surpassed the approaches reported in the state of the art. Their corresponding hyperparameter sequences are depicted in Figure 5 by means of zooming into Figure 4.

From Figure 5, it can be deduced that the networks with the best performance are those trained with the Adam optimizer at a learning rate of 0.0001. Furthermore, from the 135 configurations surpassing the architectures reported in the state of the art, 13 of them, which are shown in Table 2, achieve 100% accuracy, precision, recall, and F1 scores. This table shows all the tunable hyperparameter arrangements, with a 0.0001 learning rate, as well as the scalogram computing and processing times, the corresponding architecture training time, and the total number of parameters for each network configuration. The mean square error (L2) was used as the second selection criterion for these 13 configurations, and the number of parameters required by the network was the third one. Hence, the second configuration in Table 2, highlighted in bold, was selected as the best network composition.

### 5.2. PIN Architecture and Comparison

**Figure 6:**
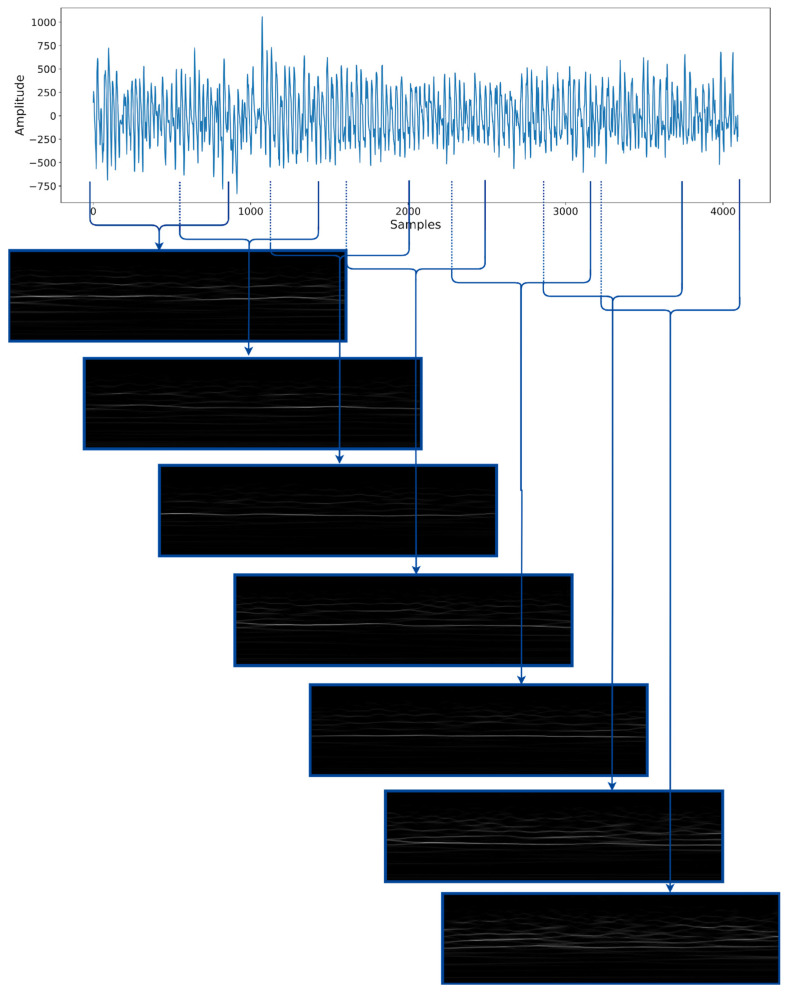
Original signal sampling and its transformation into scalograms as inputs for the PIN.

The network arrangement with the best performance was designated as parallel ictal-net (PIN), which was configured with the following hyperparameters: (a) the bump wavelet mother function, (b) without an assigned sampling frequency, (c) a window extent of 868 samples, (d) with a 33% overlap between consecutive windows, as shown in Figure 6, and (e) a learning rate of 0.0001. The scalogram computation elapsed for 26.04 s, the training time was 352.78 s for the 10 trials, and the L2 function value was 0.47.

Figure 6 illustrates that the input for the parallel ictal-net (PIN) consisted of seven scalograms, each with 868 samples analyzed across 233 scales, resulting in dimensions of 233 × 868 × 7. To manage the network’s computational load and prevent memory overflow, these inputs were resized to 250 × 250 × 7 using a bilinear interpolation, except for smaller windows. This resizing reduced the number of input parameters.

Each scalogram input into the PIN was processed through seven convolutional blocks. At the beginning of each block, a 2D layer with 10 filters applied a 3 × 3 mask, forwarding the processed data to a 2D efficient channel attention module. The data then passed through an ReLU activation function and a max-pooling layer with a 2 × 2 kernel.

After the convolutional blocks, a flattening layer adjusted the dimensions of the extracted features. These features were then classified using a dense layer with two output neurons employing a SoftMax activation function.

**Figure 7:**
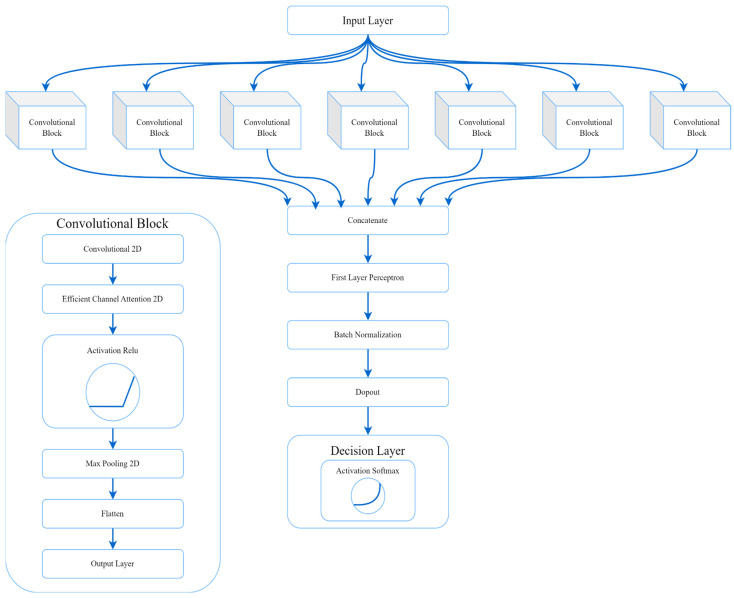
Architecture of the parallel ictal-net model.

The outputs from the seven convolutional blocks were merged and passed through a dense layer with 432 neurons and an ReLU activation function. This was followed by the batch normalization stage, a dropout layer set at a 0.7 rate, and a final two-neuron dense layer with a SoftMax activation function. Figure 7 presents a flowchart of the PIN architecture, offering a clear visual representation of this process.

Table 3 shows a comparative performance analysis of the proposed PIN against recently introduced approaches in the state of the art about identifying ictal and interictal states, taking as benchmarks the following four performance metrics: accuracy, precision, recall, and F1 scores. However, it is worth noting that not all the methods in the literature report the F1 score. From this table, it can be observed that the proposed PIN surpasses the previous proposals in the state of the art by at least 1.14%, taking into consideration all the analyzed performance metrics.

**Figure 8:**
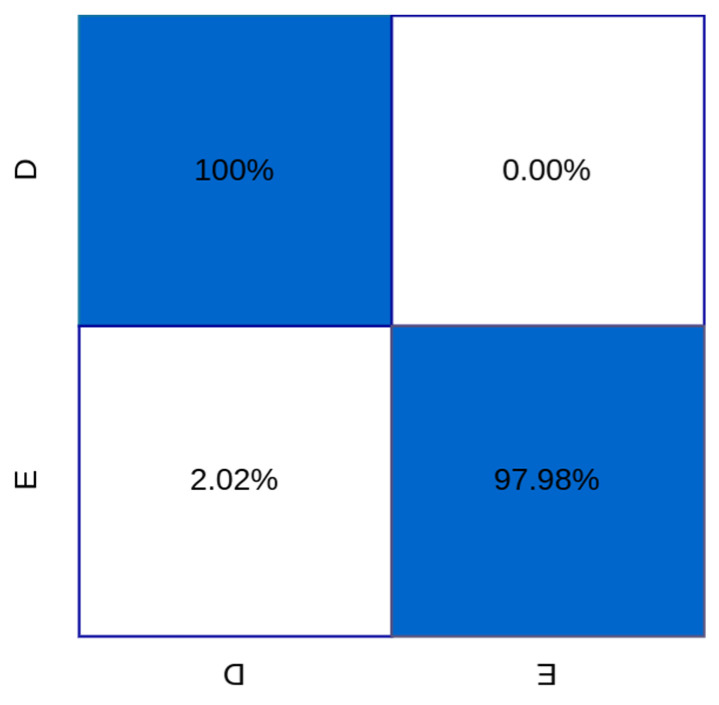
Confusion matrix for the classification of subsets D and E using the PIN architecture.

In Figure 8, the confusion matrix for the classification of subsets D and E in the University of Bonn database is presented. These values were utilized to derive the metrics reported in Table 3.

## 6. Conclusions

Epilepsy is a neurological disorder that not only restricts those who suffer from it, but also affects their families, making them targets of social discrimination. A reliable tool for the timely diagnosis of epilepsy can help mitigate the social and emotional impacts on patients. Electroencephalography (EEG) signals provide physiological indicators that are crucial for diagnosing, treating, and tracing epilepsy. Consequently, several works are dedicated to analyzing the publicly available database from Bonn University. However, the reliable classification of ictal and interictal states from EEG signals taken in the epileptogenic zone remains an issue. Therefore, in this work, we performed a thorough analysis of various neural network configurations for classifying ictal and interictal events. Our results show that 135 out of 2880 different CNN arrangements surpass recently proposed approaches in the reviewed literature. Furthermore, 13 of these 135 structures demonstrate 99% certainty in detecting ictal and interictal stages, considering the reliability metrics of accuracy, precision, recall, and F1 scores. Moreover, the network architecture identified in Table 1 as parallel ictal-net (PIN) was selected due to the number of parameters required to configure it and its low mean square error (L2). The PIN leverages efficient channel attention modules within its parallel convolutional blocks to enhance its performance, analyzing EEG signal scalograms derived via the continuous wavelet transform (CWT), implemented using the ssqueezepy library in Python. This approach not only yielded high accuracy results, but also optimized the processing speed, paving the way for potential online applications for epilepsy diagnosis and monitoring.

## Figures and Tables

**Table 1:** Hyperparameters.

Hyperparameters	Values
Wavelet	gmw, morlet, bump, cmhat, hhhat
Fs	None, 173.61
Samples per window	174, 347, 521, 694, 868, 1042, 1215, 1389, 1562, 1736, 1910, 2083
Percent of overlap	0, 0.25, 0.33, 0.50
Learning rate	0.01, 0.001, 0.0001
Optimizer	Adam, SGD *

SGD * stochastic gradient descent.

**Table 2:** Best networks resulting from the PIN.

Wavelet	Fs [Hz]	Samples per Window	Percent of Overlap [%]	Time Scalograms [s]	Time Training [s]	L2	Number of Parameters
gmw	None	1042	33	12.67	236.90	0.47	7,309,640
**bump**	**None**	**868**	**33**	**26.04**	**382.78**	**0.47**	**6,981,109**
morlet	173.61	1042	25	11.59	265.73	0.49	6,143,871
cmhat	173.61	1042	33	24.23	515.29	0.50	8,994,440
bump	None	1042	00	14.65	322.52	0.51	4,790,902
bump	173.61	1215	00	14.93	345.51	0.51	5,582,102
bump	173.61	1736	25	11.27	285.61	0.51	6,196,313
cmhat	173.61	1389	50	19.48	427.03	0.52	9,987,071
morlet	173.61	1736	00	7.62	264.83	0.52	6,352,373
bump	None	1389	00	11.02	284.53	0.53	4,787,633
cmhat	None	1389	25	16.60	391.85	0.53	7,990,262
gmw	None	2083	25	6.01	189.41	0.53	7,556,333
gmw	173.61	2083	33	6.01	181.66	0.54	7,556,333

Values in bold indicate the variables of the best-scoring network.

**Table 3:** Comparison of the metrics obtained with the PIN against the best architectures found in the state of the art that perform the classifications of subsets D and E from the University of Bonn dataset.

**Classifier**	**Input**	**Transform**	**Validation Type**	*accuracy* **[%]**	*precision* **[%]**	*recall* **[%]**	F1 *score* **[%]**
Parallel ictal-net	Scalograms	CWT	10-fold cross-validation	98.99	99.09	99.00	98.99
			LOSO	99.49	99.54	99.5	99.49
			70% training 30% testing	97.99	98.18	98.00	97.99
Ictal-net [16]	Scalograms	CWT	60% training 20% validating 20% testing	93.90	95.47	92.19	93.80
RF [5]	Min. amp., mean amp., SD, PSD, max PSD, mean PSD, and var. of PSD	DWT	70% training 30% testing	97.04	97.00	97.73	-
SVM [5]	Min. amp., mean amp., SD, PSD, max PSD, mean PSD, and var. of PSD	DWT	70% training 30% testing	93.52	93.50	92.04	-
KNN [5]	Min. amp., mean amp., SD, PSD, max PSD, mean PSD, and var. of PSD	DWT	70% training 30% testing	97.96	98.00	97.35	-
DT [5]	Min. amp., mean amp., SD, PSD, max PSD, mean PSD, and var. of PSD	DWT	70 %training 30% testing	96.67	96.70	97.73	-
MLP [5]	Min. amp., mean amp., SD, PSD, max PSD, mean PSD, and var. of PSD	DWT	70% training 30% testing	98.15	98.20	96.59	-
FURIA [5]	Min. amp., mean amp., SD, PSD, max PSD, mean PSD, and var. of PSD	DWT	70% training 30% testing	96.30	96.30	97.73	-
FLR [5]	Min. amp., mean amp., SD, PSD, max PSD, mean PSD, and var. of PSD	DWT	70% training 30% testing	93.15	93.30	96.21	-
FRNN [5]	Min. amp., mean amp., SD, PSD, max PSD, mean PSD, and var. of PSD	DWT	70% training 30% testing	98.70	98.70	98.86	-
VQNN [5]	Min. amp., mean amp., SD, PSD, max PSD, mean PSD, and var. of PSD	DWT	70% training 30% testing	97.59	97.60	97.73	-
FNN [5]	Min. amp., mean amp., SD, PSD, max PSD, mean PSD, and var. of PSD	DWT	70% training 30% testing	96.85	96.90	98.84	-
CNN [19]	Scalograms	CWT	10-fold cross-validation	98.50	-	98.98	98.50
LSTM [15]	-	-	10-fold cross-validation	95.00	-	91.00	-
SVM [6]	Mean, variance, SD, RMS, mean PSD	RBF PSD	2-fold cross-validation	88.60	-	94.47	-
SVM [6]	Mean, variance, SD, RMS, mean PSD	RBF PSD	4-fold cross-validation	88.80	-	94.60	-
KNN [6]	Mean, variance, SD, RMS, mean PSD	RBF PSD	2-fold cross-validation	89.23	-	89.37	-
KNN [6]	Mean, variance, SD, RMS, mean PSD	RBF PSD	4-fold cross-validation	88.86	-	89.96	-
BN [6]	Mean, variance, SD, RMS, mean PSD	RBF PSD	2-fold cross-validation	91.16	-	91.37	-
BN [6]	Mean, variance, SD, RMS, mean PSD	RBF PSD	4-fold cross-validation	90.39	-	90.69	-
SVM [12]	Mean, variance, skewness, and kurrtosis	EMD	60% training 40% testing	96.25	-	-	-
SVM [12]	Coefficients A and D	DWT	60% training 40% testing	96.25	-	-	-
KNN [12]	Mean, variance, skewness, and kurrtosis	EMD	60% training 40% testing	93.75	-	-	-
KNN [12]	Coefficients A and D	DWT	60% training 40% testing	95.00	-	-	-
DT [12]	Mean, variance, skewness, and kurrtosis	EMD	60% training 40% testing	97.50	-	-	-
DT [12]	Coefficients A and D	DWT	60% training 40% testing	98.75	-	-	-
CNN FE [14]	GASF	GAF	80% training 20% testing	94.50	-	99.00	-
CNN FE [14]	GADF	GAF	80% training 20% testing	94.00	-	99.00	-
CNN IP [14]	GASF	GAF	80% training 20% testing	96.50	-	97.00	-
CNN IP [14]	GADF	GAF	80% training 20% testing	97.00	-	97.00	-

The shaded cells highlight the values that rate the performance of the proposed architecture. The symbol ‘-’ indicates that no information is available. SD: standard deviation; PSD: power spectral density; EMD: empirical mode decomposition; LOSO: leave one subject out.

## Data Availability

Publicly available datasets were analyzed in this study. This data can be found here: https://www.ukbonn.de/epileptologie/arbeitsgruppen/ag-lehnertz-neurophysik/downloads/.

## References

[1] Fisher R.S., Acevedo C., Arzimanoglou A., Bogacz A., Cross J.H., Elger C.E., Engel J., Forsgren L., French J.A., Glynn M. (2014). ILAE Official Report: A Practical Clinical Definition of Epilepsy. Epilepsia.

[2] Shoeibi A., Khodatars M., Ghassemi N., Jafari M., Moridian P., Alizadehsani R., Panahiazar M., Khozeimeh F., Zare A., Hosseini-Nejad H. (2021). Epileptic Seizures Detection Using Deep Learning Techniques: A Review. Int. J. Environ. Res. Public Health.

[3] Chou C.-H., Shen T.-W., Tung H., Hsieh P.F., Kuo C.-E., Chen T.-M., Yang C.-W. (2023). Convolutional Neural Network-Based Fast Seizure Detection from Video Electroencephalograms. Biomed. Signal Process. Control.

[4] Rafid Ahmad S.R., Sayeed S.M., Ahmed Z., Siddique N.M., Parvez M.Z. Prediction of Epileptic Seizures Using Support Vector Machine and Regularization. Proceedings of the 2020 IEEE Region 10 Symposium (TENSYMP).

[5] Aayesha, Qureshi M.B., Afzaal M., Qureshi M.S., Fayaz M. (2021). Machine Learning-Based EEG Signals Classification Model for Epileptic Seizure Detection. Multimed. Tools Appl..

[6] Kumari R.S.S., Abirami R. Automatic Detection and Classification of Epileptic Seizure Using Radial Basis Function and Power Spectral Density. Proceedings of the 2019 International Conference on Wireless Communications Signal Processing and Networking (WiSPNET).

[7] Xu X., Lin M., Xu T. (2022). Epilepsy Seizures Prediction Based on Nonlinear Features of EEG Signal and Gradient Boosting Decision Tree. Int. J. Environ. Res. Public Health.

[8] Daoud H., Bayoumi M.A. (2019). Efficient Epileptic Seizure Prediction Based on Deep Learning. IEEE Trans. Biomed. Circuits Syst..

[9] Muhammad Usman S., Khalid S., Bashir S. (2021). A Deep Learning Based Ensemble Learning Method for Epileptic Seizure Prediction. Comput. Biol. Med..

[10] Dissanayake T., Fernando T., Denman S., Sridharan S., Fookes C. (2021). Deep Learning for Patient-Independent Epileptic Seizure Prediction Using Scalp EEG Signals. IEEE Sens. J..

[11] Andrzejak R.G., Lehnertz K., Mormann F., Rieke C., David P., Elger C.E. (2001). Indications of Nonlinear Deterministic and Finite-Dimensional Structures in Time Series of Brain Electrical Activity: Dependence on Recording Region and Brain State. Phys. Rev. E.

[12] Bekbalanova M., Zhunis A., Duisebekov Z. Epileptic Seizure Prediction in EEG Signals Using EMD and DWT. Proceedings of the 2019 15th International Conference on Electronics, Computer and Computation (ICECCO).

[13] Nagabushanam P., Thomas George S., Radha S. (2020). EEG Signal Classification Using LSTM and Improved Neural Network Algorithms. Soft Comput..

[14] Shankar A., Khaing H.K., Dandapat S., Barma S. Epileptic Seizure Classification Based on Gramian Angular Field Transformation and Deep Learning. Proceedings of the 2020 IEEE Applied Signal Processing Conference (ASPCON).

[15] Shekokar K., Dour S., Ahmad G. Epileptic Seizure Classification Using LSTM. Proceedings of the 2021 8th International Conference on Signal Processing and Integrated Networks (SPIN).

[16] Hernández-Nava G., Salazar-Colores S., Ortiz-Echeverri C.J., López-Leyva S., Ramos Arreguín J.M., Salazar Colores S., Cabal Yepez E., Vargas Soto J.E. (2022). Ictal-Net: Un Diseño de CNN Para La Clasificación de Escalogramas de Electroencefalogramas Con Crisis Convulsivas. Diseño y Planeación Mecatrónica.

[17] Veeranki Y.R., McNaboe R., Posada-Quintero H.F. (2023). EEG-Based Seizure Detection Using Variable-Frequency Complex Demodulation and Convolutional Neural Networks. Signals.

[18] Ortiz-Echeverri C.J., Salazar-Colores S., Rodríguez-Reséndiz J., Gómez-Loenzo R.A. (2019). A New Approach for Motor Imagery Classification Based on Sorted Blind Source Separation, Continuous Wavelet Transform, and Convolutional Neural Network. Sensors.

[19] Stéphane M., Stéphane M. (2009). CHAPTER 7—Wavelet Bases. A Wavelet Tour of Signal Processing.

[20] Mashrur F.R., Islam M.S., Saha D.K., Islam S.M.R., Moni M.A. (2021). SCNN: Scalogram-Based Convolutional Neural Network to Detect Obstructive Sleep Apnea Using Single-Lead Electrocardiogram Signals. Comput. Biol. Med..

[21] Türk Ö., Özerdem M.S. (2019). Epilepsy Detection by Using Scalogram Based Convolutional Neural Network from EEG Signals. Brain Sci..

[22] Nkemelu D.K., Omeiza D., Lubalo N. (2018). Deep Convolutional Neural Network for Plant Seedlings Classification. arXiv.

[23] Widiastuti N.I. (2019). Convolution Neural Network for Text Mining and Natural Language Processing. IOP Conf. Ser. Mater. Sci. Eng..

[24] Tuo S., Chen T., He H., Feng Z., Zhu Y., Liu F., Li C. (2021). A Regional Industrial Economic Forecasting Model Based on a Deep Convolutional Neural Network and Big Data. Sustainability.

[25] Geng Z., Zhang Y., Li C., Han Y., Cui Y., Yu B. (2020). Energy Optimization and Prediction Modeling of Petrochemical Industries: An Improved Convolutional Neural Network Based on Cross-Feature. Energy.

[26] LeCun Y., Bengio Y., Hinton G. (2015). Deep Learning. Nature.

[27] Wang Q., Wu B., Zhu P., Li P., Zuo W., Hu Q. ECA-Net: Efficient Channel Attention for Deep Convolutional Neural Networks. Proceedings of the 2020 IEEE/CVF Conference on Computer Vision and Pattern Recognition (CVPR).

[28] Vaswani A., Shazeer N., Parmar N., Uszkoreit J., Jones L., Gomez A.N., Kaiser L., Polosukhin I. (2017). Attention Is All You Need. arXiv.

[29] Luong T., Pham H., Manning C.D. Effective Approaches to Attention-Based Neural Machine Translation. Proceedings of the 2015 Conference on Empirical Methods in Natural Language Processing.

[30] Wang X., Girshick R., Gupta A., He K. Non-Local Neural Networks. Proceedings of the 2018 IEEE/CVF Conference on Computer Vision and Pattern Recognition.

[31] Tursunov A., Mustaqeem, Choeh J.Y., Kwon S. (2021). Age and Gender Recognition Using a Convolutional Neural Network with a Specially Designed Multi-Attention Module through Speech Spectrograms. Sensors.

[32] Guo M.-H., Xu T.-X., Liu J.-J., Liu Z.-N., Jiang P.-T., Mu T.-J., Zhang S.-H., Martin R.R., Cheng M.-M., Hu S.-M. (2022). Attention Mechanisms in Computer Vision: A Survey. Comput. Vis. Media.

[33] Gupta A., Arunachalam S., Balakrishnan R. (2020). Deep Self-Attention Network for Facial Emotion Recognition. Procedia Comput. Sci..

[34] Shu X., Chang F., Zhang X., Shao C., Yang X. (2022). ECAU-Net: Efficient Channel Attention U-Net for Fetal Ultrasound Cerebellum Segmentation. Biomed. Signal Process. Control.

[35] Qin Z., Zhang P., Wu F., Li X. FcaNet: Frequency Channel Attention Networks. Proceedings of the 2021 IEEE/CVF International Conference on Computer Vision (ICCV).

[36] Rai D., Kerr M.P., McManus S., Jordanova V., Lewis G., Brugha T.S. (2012). Epilepsy and Psychiatric Comorbidity: A Nationally Representative Population-Based Study. Epilepsia.

[37] Muradeli J. Ssqueezepy, 2020. GitHub Repository. https://github.com/OverLordGoldDragon/ssqueezepy/.

[38] Chollet F. (2017). Deep Learning with Python.

